# Prospective observation of breast/ovarian cancer risk in BRCA1 carriers depending on serum selenium level optimized with diet

**DOI:** 10.1186/1897-4287-10-S1-A11

**Published:** 2012-01-12

**Authors:** J Lubiński, T Huzarski, A Jakubowska, J Gronwald, K Jaworska, M Muszyńska, G Sukiennicki, K Durda, C Cybulski, T Dębniak, A Tołoczko, O Oszurek, P Serrano-Fernandez, R Scott, S Narod

**Affiliations:** 1 International Hereditary Cancer Center, Pomeranian Medical University, Szczecin, Poland

## 

The aim of the study is to observe prospectively the possibility of lowering the cancer risk among BRCA1 carriers by optimizing selenium concentration in diet/organism. Results of studies performed in several centres, particularly of our own search, are strongly indicating on potential of decreasing breast/ovarian cancer risk among carriers by optimization of selenium concentration in the body. Studies will be performed on group of 1500 BRCA1 carriers. Cohort will be recruited during the first 6 months of the project. Mean length of follow-up will be 3 yrs. From all females serum will be collected for selenium analyses-at the beginning and, then, every 6 months. Participants will receive the list of products with selenium concentration estimated according to literature data and, additionally, information about e-store (http://www.dietaantyrakowa.pl) specialized in distribution of food products with defined amount of selenium. Information on optimal selenium concentration according to existing data will be provided also. It is expected that among ~750 carriers following recommended diet changes 38 cancers will be diagnosed and among the others ~750-60. The difference between groups will be statistically significant with p=0.0278. If necessary, investigation will be extended.

